# Building Research Capacity in Medical Radiation Sciences Through University–Clinical Partnerships: A Commentary on Working Smarter, Not Harder

**DOI:** 10.1002/jmrs.70120

**Published:** 2026-09-03

**Authors:** Therese Gunn, Michael Neep, Pamela Rowntree, Deborah Starkey

**Affiliations:** ^1^ School of Clinical Sciences Queensland University of Technology Brisbane Australia; ^2^ Department of Medical Imaging Logan Hospital Meadowbrook Queensland Australia

## Abstract

A research partnership model frames university–clinical collaboration as central to strengthening medical radiation sciences research capacity. By aligning students, academics, clinicians, resources, ethics processes, skills development and outputs, the model supports sustainable, evidence‐informed practice with potential benefits for patients and professionals.

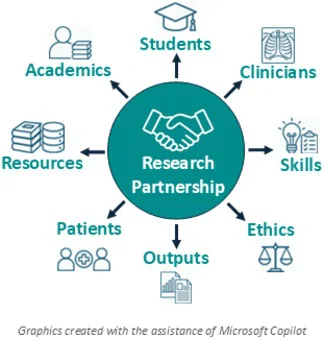

*‘I am neither especially clever nor especially gifted. I am only very, very curious.’*

*Albert Einstein* [1]

Building research capacity in Medical Radiation Sciences (MRS) is essential to evidence‐based practice and improved patient outcomes. This commentary proposes a collaborative university‐clinical model, beginning at the undergraduate level, to foster sustainable research engagement. Using the Queensland University of Technology (QUT) embedded Honours programme as an example, the authors highlight how structured student research, supported by academic mentorship and university resources, can develop foundational research skills and promote curiosity among future MRS professionals.

By linking student projects with clinical partners, departments gain access to motivated researchers, while students gain real‐world experience and mentorship. This model addresses barriers such as time, confidence and access to resources, reframing them as requirements for successful research. Data from national conference presentations by recent graduates demonstrate increased dissemination and ongoing engagement.

The commentary encourages a shift in mindset: from viewing research barriers to recognising it as achievable through collaboration. It calls for partnerships that support mutual growth, elevate professional standards, and enhance patient care. Academic‐clinical partnerships through the Honours programmes may offer a practical pathway to strengthen MRS research capacity.

Multiple publications highlight the benefits, enablers, and barriers to developing a sustainable research culture within the medical radiation science (MRS) professions [2, 3, 4, 5, 6]. Evidence also suggests that improving research capacity among health professionals is required to meet professional standards [7, 8, 9, 10, 11]. While barriers have been discussed extensively, this commentary focuses on where research culture should begin: at the undergraduate level. Collaboration between university academics and clinical departments is already valued through clinical placements; extending this collaboration to research is a logical next step. Academics have access to librarians, statisticians, ethics advisors, data collection and analysis software, and institutional support for research outputs. Academics are also actively encouraged and supported to progress their own research outputs and have the capacity and skills to support others in their research goals. University programmes also have a pool of eager students who must meet Medical Radiation Practice Board of Australia (MRPBA) Professional Capabilities, including those related to research [12]. The authors propose it would be prudent that linking clinical partners with student projects can build research capacity and debunk the myth that research is difficult. The difficult part is knowing how to start.

The purpose of this commentary is to highlight how university‐clinical research collaborations, beginning with undergraduate Honours research, can build sustainable research capacity in medical radiation sciences and ultimately improve patient care.

## 1 Background

**FIGURE 1:**
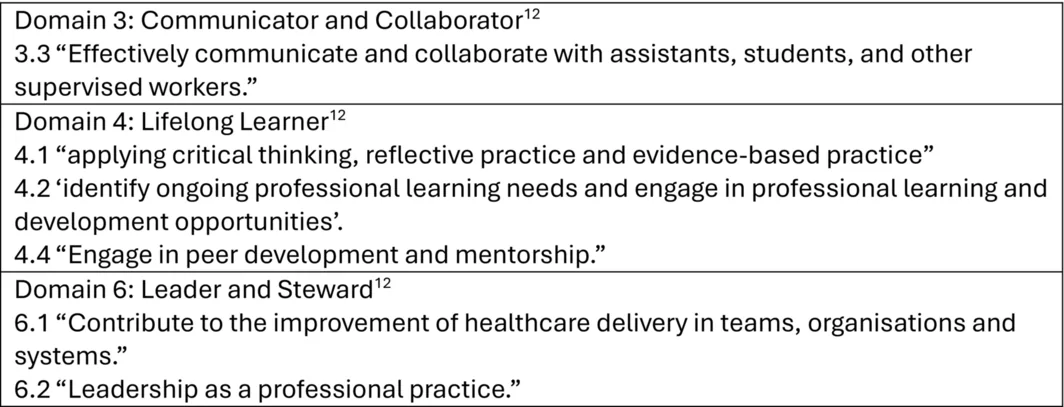
MRPBA Domains relevant to research capacity and collaboration [12].

Dennett et al., among others, have contributed significantly to investigating research capacity within MRS [2]. Evidence‐based practice improves patient outcomes through workflow efficiencies, patient safety and person‐centred care [2, 3, 6]. An extrinsic motivation for both academics and students is that all registered MRPBA professionals must meet capabilities across all domains. Equally, registered MRPs need to maintain continuing professional development aligning to the MRPBA domains [12]. Domains of significance to this commentary are demonstrated in Figure 1.

There is a professional expectation that MRS professions and all allied health professionals are ‘research literate’, engage with research in practice and generate new research [2, 13]. This challenges the traditional model of MRS participation in research led by medical practitioners such as radiologists, radiation oncologists or nuclear medicine physicians [2, 4, 6]. It is time for MRS professions to lead and value the contribution research can make within our space. Graduates enter the workforce at a novice research level after completing an Honours research project, with the hope to continue building these skills. With momentum, encouragement and guidance, early career MRS professionals could become valuable research resources to clinical departments.

A 2022 study by Angus et al. has highlighted the potential of allied health workers supervising student research projects to contribute to healthcare performance, research capacity and personal research achievements [14]. Students also gain collaboration and mentorship skills, reinvigorating university‐clinical partnerships beyond clinical placements [14]. This commentary proposes that enhancing fundamental research skills in undergraduate curricula gives future MRS professionals the critical thinking and problem‐solving skills needed to contribute to clinical research teams [14].

The undergraduate MRS pathway incorporating an embedded Honours degree is offered at multiple universities and provides a foundation in research skills development. Graduates enter the profession with experience in structuring a research project, finding and appraising literature, and hopefully, a thirst for curiosity. Workplaces with research opportunities and supported pathways can enable early dissemination at state or national conferences or by publication [14]. Undergraduate students have access to university resources, mentors/supervisors and dedicated training workshops to develop research skills. All undergraduate Australian and New Zealand MRS degrees promote research skill development, with the Honours pathway being more identifiable. Graduates entering the workforce with research skills should be considered a source of knowledge ready to contribute to any MRS department.

Stair et al.'s [15] study described a structured approach to engaging students with research throughout a degree, culminating in a capstone project to enhance research capabilities [13]. This commentary discusses this approach through the authors' knowledge of one university Honours programme and its research outcomes over several years.

At the example university, the QUT final year students attend facilitated on‐campus workshops focused on fundamental research skills. These culminate in two major pieces of assessment in the second semester: a seminar for clinical partners, with oral presentations in a 3‐min thesis format; and a written manuscript following the Journal of Medical Radiation Sciences (JMRS) author guidelines. After submission and grading, students are encouraged to work with their supervisors as co‐authors to progress their manuscripts towards publication.

Table 1 provides a sample of workshop topics, skills and the structure of the QUT two‐semester, 24 credit point unit, including learning outcomes:

**TABLE 1:** Learning outcomes and sample workshop content covered in the Honours' project capstone units.

Learning outcomes	Research skills explicitly taught
On successful completion of this unit, you will be able to: Apply research skills to design, plan and execute a research project. Select and critically analyse peer reviewed literature relevant to your topic. Collect, organise and analyse data and interpret findings. Report research findings through written and other methods to peers and medical imaging professionals.	Library skills—writing a PICO question; effective search strategies; setting up alerts for articles; ethical use of generative AI.
	Selecting quality papers for review; critical appraisal
	Ethics—history, importance, process, governance, value of research teams.
	Peer review—feedback and responding to feedback.
	Value of disseminating research; examples of Medical Imaging research in practice and previous student success.
	How to write a journal article.

Abbreviation: PICO = Population/Problem, Intervention, Comparison, Outcome.

## 2 Requirements for Successful and Sustainable Research (Formally Known as ‘Barriers’)

Rather than focusing on long reported barriers preventing MRS clinicians from participating in research, what if the language and mindset shifted to the requirements for successful and sustainable research? Each requirement can then be considered in terms of how university‐clinical collaboration could build research capacity in MRS.

Mdletshe, 2023, offered multiple solutions to enhance MRS research culture, including university driving research [16]. This commentary continues that conversation by highlighting the benefits of collaboration between universities and clinical departments. Student projects based on clinical partner ideas can reduce some time barriers. The student and academic supervisors can absorb some of the time‐consuming tasks, such as literature reviews and ethics applications. Universities can directly support confidence and mentoring through access to wider resources and an existing research culture.

While this commentary only provides one university example in the MRS research space, academic research can enhance clinical research culture only through genuine partnerships that benefit all. Such synergy can benefit the professional community, patients, health outcomes and the community we serve [7, 8].

Table 2 highlights requirements, formally known as ‘barriers’ from the MRS literature in this space. Using evidence from the literature and the authors' collective knowledge of an example university Honours programme, suggestions are provided on how a collaborative approach could form a basis of achieving some of these requirements.

**TABLE 2:** Requirements for successful and sustainable research and proposed solutions.

Requirements for successful and sustainable research from relevant literature	Capacity of how research partnerships between universities and clinical departments aligns with the research evidence
Time [2, 3, 4, 6, 16, 17]	Students work on their research for two semesters + academics aiming to meet KPIs for research outputs = > Clinical radiographers work as content experts while learning and gaining experience without a full‐time commitment as research is driven by student.
Reduce workload pressures [2]	
Research skills [3] and knowledge [2]	Students have access to content (see Table 1); access to university library data bases and software; access to librarian support; structured project completed in 12 months. Collaboration reduces MRPs feeling of being overwhelmed in where to start, can gain experience of the process and gain confidence without needing to lead, create or produce independent research.
Access to equipment/resources [2]	
Understanding of research and ethic processes (reduced fear) [2] overwhelmed (ethics) [14]	
Leadership/workplace support [3, 4]	Graduates entering the workforce will 1 day become leaders. Sustainable encouragement will potentially provide a shift in culture and value of clinical research. Co‐authorship with a collaborative approach will increase collective outputs and have the potential to improve the departments' view and value of research.
Organisational culture [2]	
Incentives [2]	Students complete the literature review/gap to provide evidence and information to inform grant applications; Academics have access to grant writing workshops and a network of Chief Investigators; Clinicians value research, co‐authorship of outputs and potential immediate quality improvement outcomes—impact may lead to grant success.
Financial support [4, 14]	
Access to mentors [9, 17]	All undergraduate students have access to an academic supervisor. If the original idea is from a clinical site, the MRP will form part of the supervisory team; novice research clinicians and academics gain experience before embarking on HDR supervision.
Interest in research [4, 14] Motivation [16]	Students have potential to have a positive research experience, guided by enthusiastic supervisors gaining experience of the research process. A positive experience will potentially build momentum to continue asking questions that lead to research projects.

## 3 Sustainable Research Practice

Embedded Honours pathways across Australian and New Zealand university degrees create opportunities to deepen research engagement and equip graduate radiographers with confidence to remain curious in the clinical setting. This may extend to higher degree research at a Masters or PhD level, or through valuable smaller‐scale studies.

Projects may be led by universities, students, academics or clinical partners. Experienced clinical staff or research‐intensive clinical leads may be able to offer part of a larger trial or project to an Honours student, enabling the student to learn within a broader research team. Likewise, a clinician new to research may contribute an idea for a student to investigate while learning the process alongside the student, supported by academics. As McNulty discussed, MRS research outputs are increasing, but quality must remain central [6]. Iwika et al. concluded that studies at the peak of research hierarchy remain limited [18]. Strengthening the MRS evidence base requires more systematic reviews, randomised control trials, and multi‐site experimental studies [18]. The authors are not suggesting a nine‐month Honours research project will produce high‐level research outcomes or major practice change. However, these projects could inform clinical decisions through audits or quality approval projects, provide a foundation for future studies and train students in research thinking. It may also offer:

- A quest for knowledge and future impactful research
- A pilot or audit within a wider project to inform future studies or grants
- Collaborations that support ongoing, mutually beneficial university‐clinical relationships.
- Reduced fear of research and ethics processes

## 4 Case Studies—MI Honours Programme at QUT

For example, in the QUT Medical Imaging programme, students propose topics for projects that are either of an experimental/quality improvement/audit nature or as a narrative review. An invitation is sent to the clinical placement sites in search of potential projects or curiosities in the clinical space that could provide an avenue of research as options for the students. The students have an option to select one of these ideas or nominate their own topic. Any topic provided by a clinical radiographer will offer an opportunity to be a co‐supervisor in partnership with a university academic. The workload required of the clinical partner varies according to the complexity of the project and their existing knowledge of the subject matter. The aim of this partnership is to publish a manuscript with all contributors listed as authors; anecdotal feedback from clinical partners suggests that this model reduces their workload compared with undertaking the research independently.

### 4.1 Example Categories of Previous Honour's Projects

1. Narrative review—a topic may be suggested from a clinical partner, and a search of the literature is done by the student. The time frame is not conducive to a systematic (or scoping) review; however, it may provide background information for a larger project or grant application. Examples of presentations are by Jong et al. [19] and Lim et al. [20].
2. Experimental study—performed at university simulation laboratory (no ethics required). This again could be based on a clinical suggestion. Historically, students have evaluated technical parameters, including dose, anode/heel effect, Source Image Distance impact. Examples of published experimental papers are by Keir and Braithwaite [21], Searl and Starkey [22] and MacDonald et al. [23].
3. Clinical partnership (experimental, audit, Quality Assurance (QA) projects)—Clinical partners may have had existing data that was part of a previous project that could provide a focus on a sub‐set for analysis or be able to retrospectively analyse existing data for QA or audit purposes. If an existing project, a variation to the ethics application is required as well as an administrative review from the university as a partner. This is generally a quicker option than creating a new ethics application. New projects requiring ethics approval from both the clinical department and the university (administrative review) can be daunting at first and require time and commitment from the clinical lead researcher for site specific processes. After an application has been completed once, supervisor knowledge improves and each project gets easier. As the ethics process varies, the general time for the ethics approval to be granted is between 1 and 5 months. The administrative review process from the university once the project has site specific approval has been straightforward. The authors have had success in working with specific hospitals and their research teams making the process more streamlined, the more we collaborate. Examples of published experimental papers aligned with clinical partners are by Atkinson et al. [24] and Poon et al. [25].

Students are encouraged to disseminate their findings via various methods. During the final semester, they are invited to submit an abstract for the ASMIRT student paper night, followed by abstracts to present either an oral or poster presentation at the following ASMIRT national conference, and/or publish in an appropriate journal.

To provide evidence that the embedded Honour's programme at QUT increases research outputs, the authors of this commentary reviewed the published abstracts in JMRS of the Australian Society of Medical Imaging Radiation Therapy (ASMIRT) national conferences of both the oral and poster presentations for the years 2021–2025. Focus was on any authors that were able to be identified as graduates from within the 5 years prior to publication. As this was publicly available data, no ethics was required. Data was categorised as graduates presenting within 5 years of completing the university degree. The research project may or may not have been related to the undergraduate project and may have been a supported clinical project. See Table 3 for descriptive results per year from 2021 to 2026 for graduates presenting either oral or poster presentations at ASMIRT, as well as current student participation. This data demonstrate an increase in participation rates in professional dissemination of research projects by newly qualified MI graduates, hopefully leading to continued interest and motivation within the research space. Additionally, over the past 5 years, the publication rate of Honours students following completion has been low, with a conversion rate of 2.8% in the 316 graduates from 2020 to 2024. The authors hope this figure will increase as relationships with clinical partners continue to strengthen and students become more aware of the value of engaging in research during their undergraduate studies.

**TABLE 3:** Recent graduate numbers participating in either oral or poster presentations or publications.

Year	Number of presentations (either an oral or poster) within 5 years of graduation	Number of publications
2021	2	3 [26, 27, 28]
2022	9	—
2023	8	2 [21, 29]
2024	10	3 [23, 30, 31]
2025	12	1 [25]
2026	5	—
TOTAL	46	9

## 5 Limitations

This commentary is from the perspective of the authors as medical imaging academics from only one university programme. Assumptions are made that programmes within the MRS professions and at other Australian and New Zealand institutions would offer similar graduate research skill sets and have a willingness to form collaborative partnerships with clinical researchers.

## 6 Conclusion

The more exposure students and ultimately radiographers gain by participating in research, no matter how small or large, informal or formal, the collective knowledge and expertise of the profession will be elevated. The authors propose that encouraging university and clinical collaboration will be a success story by providing a supportive way to include clinical radiographers in research projects with university partners. University collaboration can provide access to research skills, literature, resources, ethics and governance support and research software. Future research should examine the long‐term sustainability of this partnership model, including whether it strengthens research capacity, increases publication output and supports meaningful improvements in clinical practice and patient care. To evaluate, we need to create the partnerships, complete the research projects and slowly build the capacity. A review of the partnerships through an analysis of clinical partner feedback and discussions will be required to determine if this is a successful framework to build capacity in MRS research. In the words of Kev Carmody and Paul Kelly, ‘From little things, big things grow’ [32]. It is now up to each of us to make a start, join forces and work together for the common goal to benefit the MRS professions and ultimately the patients.

## Funding

The authors have nothing to report.

## Ethics Statement

The authors have nothing to report.

## Conflicts of Interest

The authors declare no conflicts of interest.

## Data Availability

The authors have nothing to report.
